# Association of Fatigue With Sleep Duration and Bedtime During the Third Trimester

**DOI:** 10.3389/fpsyt.2022.925898

**Published:** 2022-07-06

**Authors:** Duo Ma, Yimin Kang, Denglan Wang, Haoxiong Chen, Ligang Shan, Chun Song, Yanlong Liu, Fan Wang, Hui Li

**Affiliations:** ^1^Department of Ultrasonography, The Second Affiliated Hospital of Xiamen Medical College, Xiamen, China; ^2^Key Laboratory of Psychosomatic Medicine, Inner Mongolia Medical University, Huhhot, China; ^3^Xinjiang Key Laboratory of Neurological Disorder Research, The Second Affiliated Hospital of Xinjiang Medical University, Ürümqi, China; ^4^Department of Obstetrics, The Second Affiliated Hospital of Xinjiang Medical University, Ürümqi, China; ^5^Department of Anesthesiology, The Second Affiliated Hospital of Xiamen Medical College, Xiamen, China; ^6^School of Mental Health, Wenzhou Medical University, Wenzhou, China; ^7^The Affiliated Kangning Hospital, Wenzhou Medical University, Wenzhou, China; ^8^Beijing Hui-Long-Guan Hospital, Peking University, Beijing, China; ^9^Department of Biomedical Engineering, College of Future Technology, Peking University, Beijing, China

**Keywords:** bedtime, fatigue, pregnant, sleep duration, third trimester

## Abstract

**Purpose:**

To investigate the association between fatigue and sleep habits of pregnant women to further explore the effect of sleep duration and bedtime on fatigue during the third trimester.

**Materials and Methods:**

A total of 465 Chinese Han pregnant women in the third trimester (after 28 weeks) with a singleton gestation were recruited. Sleep habits (such as bedtime, sleep onset latency, and night sleep duration) and the 14-item Fatigue Scale scores (FS-14, used to assess fatigue) were collected.

**Results:**

The effects of sleep duration and bedtime on FS-14 physical and total scores were significant. FS-14 physical scores and total scores of the participants in the group of sleep before 23 o’clock (SBC) of short sleep duration (<7 h) were significantly higher as compared to the participants in the group of SBC of normal sleep duration, and those of the participants in the group of SBC of normal sleep duration were significantly lower than the participants in the group of sleep after 23 o’clock of normal sleep duration. There were negative correlations of sleep duration with FS-14 physical score and total score in the SBC of short sleep duration group.

**Conclusion:**

Sleep less than 7 h or bedtime after 23 o’clock was associated with increased fatigue levels of pregnant women in the third trimester. Therefore, it is necessary to develop good sleep habits (enough sleep duration and early bedtime) to keep fatigue at a low level for pregnant women in the third trimester.

## Introduction

Fatigue as a common discomfort symptom during pregnancy may not only reduce the quality of life but also contribute to severe outcomes, such as severe labor pain, postpartum depression, and cesarean deliveries (1, 2), and can adversely affect fetal heart rate and produce fetal distress, increasing the incidence of instrumental and cesarean births. In addition, fatigue may be associated with a decrease in the mother’s interest in the newborns, “baby blues” and postpartum depression, compromising the mother-baby bond (2–4). Insufficient sleep duration is always common during pregnancy (5), and it may increase fatigue and the risk of adverse pregnancy outcomes (6).

It has been reported that short sleep duration during the third trimester was associated with intense fatigue in Chinese women (7), while Chinese women in the third trimester of pregnancy endured a higher degree of fatigue than those in the first and second trimesters based on a longitudinal study (8). A consensus statement suggests that, in general, adults should have no less than 7 h of sleep per night (9). Less than 7 h of sleep per night, as one kind of short sleep duration, leads to increased reporting of fatigue (10) and is associated with adverse health outcomes (9, 11, 12). Nevertheless, the third trimester is characterized by a decrease in sleep duration (6), which is easily linked to fatigue.

Approximately 60% of pregnant women during the third trimester report being fatigued (13). Total sleep time reduction during the third trimester was related to fatigue levels (14). A prospective study of pregnant women found that the self-reported sleep quantity was associated with reported fatigue levels (15). Increased fatigue is associated with self-reported sleep disturbances and an inverse association has been reported between fatigue and self-reported nocturnal sleep duration during the third trimester of pregnancy (16, 17). Fatigue in early labor was reported even to be negatively correlated with total nocturnal sleep duration (18). Although these findings result from varying conceptualizations, definitions, and estimates of sleep and fatigue during the third trimester, they all indicate that fatigue in the trimester is real and significant and is closely associated with the sleep-related time.

Fatigue, as one symptom of the delayed sleep phase, is consistent with insufficient sleep (19). While late bedtime, defined as a bedtime after 11:00 p.m. (20), is closely associated with insufficient sleep (21), people who habitually go to sleep late have a delayed sleep phase (22), which is included in circadian rhythm disturbances (23). Young adults who sleep late are more likely to have sleep problems as compared to those who do not go to sleep late (24).

In modern society, late bedtime and insufficient sleep remain both common and it is difficult to disentangle the independent effects of sleep duration and bedtime on fatigue, as short sleep duration can frequently be linked to a delay in bedtime (25). Moreover, much of the existing literature focuses on insufficient sleep/sleep deprivation and its effects, but there are few studies of late bedtime on pregnant fatigue. Therefore, the present study was conducted to investigate the association between fatigue and sleep habits of pregnant women in the third trimester to further explore the effect of sleep duration and bedtime on fatigue during the third trimester.

## Materials and Methods

### Participants

A total of 465 Chinese Han pregnant women in the third trimester (after 28 weeks) with a singleton gestation were recruited. Participants’ sleep was assessed after 28 weeks.

The inclusion criteria were as follows: healthy pregnant women aged >20 years old, in the third trimester, and experiencing no pregnancy complications. Gestational age was determined from the last menstrual cycle and was verified using ultrasound scan measurements. According to medical records and self-reported data, women who worked night shifts, those who had been diagnosed with depression, those with major longstanding sleep issues and diagnosed with sleep disorders, or those who took medications and healthcare products known to affect sleep patterns were excluded. The exclusion criteria also included no history of any substance abuse or dependence and any diagnosed neurological and psychiatric disorders.

Sociodemographic data, such as age, years of education, occupation, prepregnacy details, and current body mass index (BMI), were collected. Clinical data, such as the history of substance abuse and dependence, were obtained according to medical records and self-reports and confirmed by the next of kin and family members. Sleep habits, such as drinking coffee or tea at night, bedtime, sleep onset latency, and night sleep duration, were recorded.

Participants who had a sleep duration of no more than 7 h were designed for the short sleep duration group (*n* = 163) and those who had a sleep duration of more than 7 h were designed for the normal sleep duration group (*n* = 302). Participants who sleep before 23 o’clock and who sleep after 23 o’clock were grouped into the sleep before 23 o’clock (SBC) group and the sleep after 23 o’clock (SAC) group, respectively. Of participants in the short sleep duration group, 106 participants were included in the SBC group and 57 participants in the SAC group. Of participants in the normal sleep duration group, 245 participants were included in the SBC group and 57 participants in the SAC group.

The present study was approved by the Institutional Review Board of the second affiliated hospital of Xiamen medical college and was performed in accordance with the Declaration of Helsinki, and written informed consent was obtained.

### Assessments and Laboratory Tests

In the present study, the 14-item Fatigue Scale (FS-14) was used to assess fatigue (26). FS-14 comprises 14 items with two dimensions: physical fatigue (items 1–8) and mental (items 9–14) fatigue. The total fatigue score obtained by adding up all items ranges from 0 to 42, with higher scores indicating more severe fatigue. The Chinese version of the FS-14 has been shown to have acceptable psychometric properties (27).

The data of high-density lipoprotein, low-density lipoprotein, alanine aminotransferase test, cholesterol, triglyceride, gamma-glutamyl transferase, and aspartate aminotransferase were obtained from routine tests of the subjects to evaluate the physical condition in the third trimester (36 weeks ± 7days). Sleep duration, sleep habit, and FS-14 were collected after the peripheral metabolic marker levels were measured on the same morning.

### Statistical Analysis

Continuous variables were summarized using means and standard deviations (SDs). The normality of all the variables was performed using the Shapiro-Wilk test, and only the distribution of high-density lipoprotein was normally distributed (*p* > 0.05). Therefore, the Mann-Whitney rank-sum test is used in Table 1. The homoscedasticity of residuals of the variances was verified in Levene’s test, and the results show that the residuals are contained in equal distribution (all *p* > 0.05); consequently, an analysis of covariance (ANCOVA) is used to compare differences in sleep duration and FS-14 between groups in Table 2. Multi-collinearity among covariates was estimated *via* tolerance and variance inflation factor (VIF) as the cutoff recommended thresholds for tolerance <0.1 and VIF >10 (28). Partial correlation analysis was performed to test the correlation between sleep duration and FS-14.

**TABLE 1:** The differences in clinical characteristics between groups.

Variables	Short sleep duration (≤7 h)		Normal sleep duration (>7 h)		
	SBC (≤23) (*n* = 106)	SAC (>23) (*n* = 57)	SBC (≤23) (*n* = 245)	SAC (>23) (*n* = 57)	*P*
	Mean ± SD	Mean ± SD	Mean ± SD	Mean ± SD	
Age	29.71 ± 4.07	29.84 ± 4.50	29.09 ± 3.99	27.49 ± 3.77	0.020*
Education years	13.31 ± 2.84	12.67 ± 2.97	11.90 ± 3.17	12.32 ± 2.78	< 0.001*
Pre-pregnant BMI	20.90 ± 3.13	20.93 ± 3020	21.46 ± 3.27	20.58 ± 2.86	0.14
Current BMI	25.06 ± 3.05	25.44 ± 3.54	25.27 ± 4.34	25.27 ± 4.34	0.26
High-density lipoprotein (mM/L)	1.78 ± 0.36	1.85 ± 0.57	1.70 ± 0.34	1.66 ± 0.36	0.008*
Low-density lipoprotein (mM/L)	3.01 ± 1.05	3.31 ± 0.97	3.22 ± 1.05	3.33 ± 1.07	0.36
Alanine aminotransferase (U/L)	14.23 ± 13.50	11.99 ± 9.05	12.44 ± 10.54	12.78 ± 7.68	0.26
Aspartate transaminase (U/L)	16.39 ± 6.43	15.13 ± 4.35	15.20 ± 4.18	15.20 ± 4.18	0.20
Gamma-glutamyl transferase (U/L)	10.94 ± 5.43	11.23 ± 7.50	11.24 ± 8.85	13.79 ± 8.69	0.92
Cholesterol (mM/L)	5.63 ± 1.37	5.9 ± 1.78	5.78 ± 1.24	5.84 ± 1.31	0.60
Triglyceride (mM/L)	2.83 ± 1.44	2.48 ± 1.12	2.73 ± 1.34	2.57 ± 1.21	0.87
Glucose (mM/L)	4.43 ± 0.37	4.57 ± 0.48	4.54 ± 0.54	4.58 ± 0.39	0.35
Uric acid (μmol/L)	302.62 ± 76.49	314.93 ± 67.5	301.01 ± 73.71	308.45 ± 67.83	0.42
Lactate dehydrogenase (IU/L)	162.02 ± 42.01	162.44 ± 26.67	155.61 ± 29.67	158.75 ± 28.67	0.20
Creatine kinase (IU/L)	62.26 ± 42.76	57.39 ± 33.10	53.54 ± 29.19	55.62 ± 31.49	0.19
Creatine kinase isoenzyme (U/L)	14.92 ± 12.27	13.37 ± 5.91	13.96 ± 6.37	14.22 ± 8.89	0.23

*SBC: Sleep before 23 o’clock; SAC: Sleep after 23 o’clock; SD: standard deviation; BMI: body mass index.*

All data were reported as mean ± SD using Mann-Whitney rank-sum tests, *p < 0.05.

**TABLE 2:** Comparison of the 14-item Fatigue Scale (FS-14) scores between short sleep duration and normal sleep duration groups in the third trimester of pregnancy.

	Short sleep duration (≤ 7 h)		Normal sleep duration (>7 h)		F_(1, 461)_	*P*	*Pa*	*Pb*
	SBC (≤23) (*n* = 106)	SAC (>23) (*n* = 57)	SBC (≤ 23) (*n* = 245)	SAC (>23) (*n* = 57)				
	Mean ± SD	Mean ± SD	Mean ± SD	Mean ± SD				
Sleep duration (hours)	6.69 ± 0.61	6.43 ± 0.82	8.32 ± 0.71	8.27 ± 0.58	2.22	0.14	–	—
FS-14 mental score	1.31 ± 1.17	1.23 ± 1.12	1.29 ± 1.17	1.39 ± 1.24	0.91	0.34	–	–
FS-14 physical score	2.81 ± 2.18	2.58 ± 2.03	2.16 ± 2.05	3.05 ± 2.26	5.65	0.018*	0.008*	0.24
FS-14 total score	4.12 ± 2.67	3.81 ± 2.50	3.45 ± 2.39	4.44 ± 2.87	5.88	0.016*	0.021*	0.21

*FS-14, fatigue scale-14; SBC, Sleep before 23 o’clock; SAC, Sleep after 23 o’clock; SD, standard deviation; ANCOVA, analysis of covariance; BMI, body mass index; df, degree of freedom.*

Data are reported as mean ± standard deviation (SD) using ANCOVA for each variable, with age, education, and current BMI as covariates. Pa: the differences between “short sleep duration and SBC” and “normal sleep duration and SBC”. Pb: the differences between “short sleep duration and SAC” and “normal sleep duration and SAC,” *p < 0.05.

In addition, general linear models (GLMs) were applied to test the significance of the interaction between sleep duration and bedtime and their effect on FS-14 scores. Age, education, and current BMI were included as cofactors in all models. The model was compared and tested using an F-statistic.

All statistical analyses were performed using IBM SPSS Statistics for Windows, Version 22.0 (IBM Corp., Armonk, NY, United States). Figures were created using GraphPad Prism version 8 (GraphPad Software Inc.). All tests were two-sided and the significance threshold was set at *p* < 0.05.

## Results

### Demographic and Clinical Characteristics

No participants were engaged in shift work or drank coffee/tea at night. All participants reported a sleep onset latency of fewer than 5 min.

The homogeneity of variance using Levene’s test was performed for sociodemographic and clinical variables (all *p* < 0.05), except for high-density lipoprotein. The Mann-Whitney rank-sum tests are used in Table 1.

Compared to participants with normal sleep duration, the ones with short sleep duration were significantly older in age, had significantly more education years, and had significantly higher high-density lipoprotein levels (29.75 ± 4.22 vs. 28.78 ± 3.99, 13.09 ± 2.90 vs. 11.98 ± 3.10, and 1.78 ± 0.35 vs. 1.69 ± 0.34), while no difference was observed in other sociodemographic and clinical characteristics between two groups (Table 1).

### Difference Analysis

The homoscedasticity of residuals of sleep duration and FS-14 was verified by Levene’s test, and the results showed that the residuals of age, education years, and current BMI were contained in equal distribution (all *p* > 0.05). Stepwise multiple regression analyses of age, education, and current BMI showed that no variable was removed from models (all tolerance >0.9 and VIF <2). Therefore, ANCOVA was used to compare differences in sleep duration and FS-14 scores between different groups (Table 2). With age, education, and current BMI as covariates, the effects of sleep duration and bedtime on FS-14 physical and total scores were significant (*F* = 5.65, *p* = 0.018 and *F* = 588, *p* = 0.016), and FS-14 physical and total scores in SBC of short sleep duration were significantly higher than those in SBC of normal sleep duration (*p* = 0.008 and *p* = 0.021, respectively).

### General Linear Models Analysis

**FIGURE 1:**
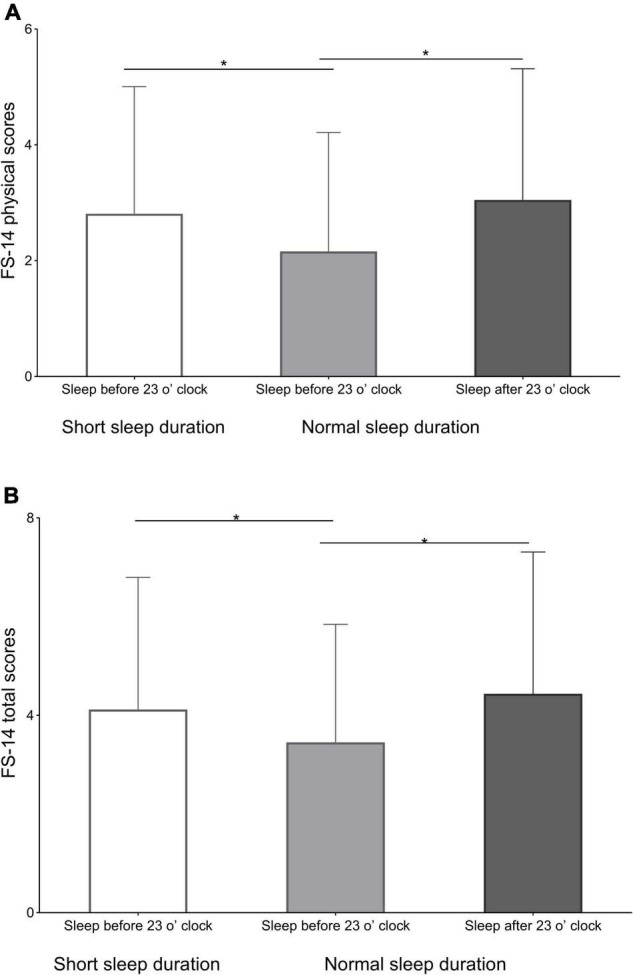
The differences in the 14-item Fatigue Scale (FS-14) physical and total scores. **(A)** The differences among the FS-14 physical scores of the participants in sleep before 23 o’clock (SBC) of short sleep duration, SBC of normal sleep duration, and sleep after 23 o’clock (SAC) of normal sleep duration. **(B)** The differences in FS-14 total scores among participants in SBC of short sleep duration, SBC of normal sleep duration, and SAC of normal sleep duration. *p < 0.05.

To test for possible interactions between the effects of sleep duration and bedtime on fatigue, the GLM analyses of FS-14 scores were performed with age, education, and current BMI as covariates. Strong interactions for physical and total scores between sleep duration and bedtime were shown by the GLM analysis in the data. The FS-14 physical scores and total scores of the participants in SBC of short sleep duration were significantly higher as compared to the participants in SBC of normal sleep duration (*p* = 0.012 and *p* = 0.034, respectively), and those of the participants in SBC of normal sleep duration were significantly lower than the participants in SAC of normal sleep duration (*p* = 0.005 and *p* = 0.011, respectively; Figure 1 and Table 3).

**TABLE 3:** The interaction of sleep duration and bedtime on the 14-item Fatigue Scale (FS-14) scores.

FS-14	Short sleep duration (≤ 7h)		Normal sleep duration (>7h)			
	SBC (≤ 23) (*n* = 106)	SAC (>23) (*n* = 57)	SBC (≤ 23) (*n* = 245)	SAC (>23) (*n* = 57)	Mean differences	*P*
Physical score	2.81 ± 2.18	–	2.16 ± 2.05	–	0.62	0.012*
		–	2.16 ± 2.05	3.05 ± 2.26	–0.88	0.005*
Total score	4.12 ± 2.67	–	3.45 ± 2.39	–	0.63	0.034*
		–	3.45 ± 2.39	4.44 ± 2.87	–0.96	0.011*

*SBC: Sleep before 23 o’clock; SAC: Sleep after 23 o’clock; GLM, general linear models.*

GLM was used to calculate the differences in levels between four groups with age, education, and BMI as covariates. The simple effect was calculated using GLM. Data were reported as mean ± standard deviation (SD), *p < 0.05.

### Correlations Analysis

**FIGURE 2:**
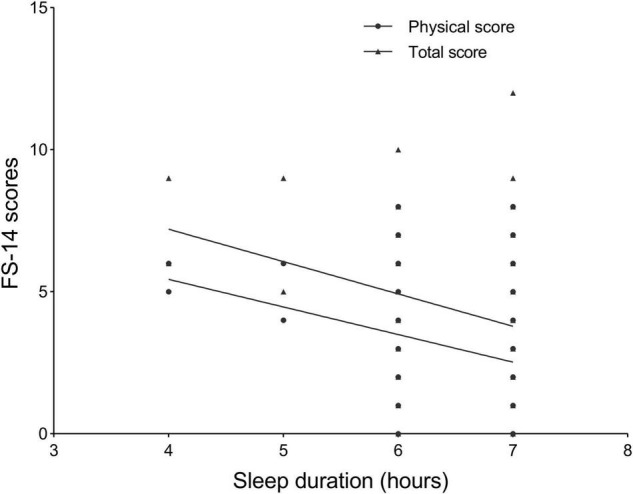
The negative correlation of sleep duration with the 14-item Fatigue Scale (FS-14) physical score (*r* = –0.319, *p* = 0.001) and FS-14 total score (*r* = –0.315, *p* = 0.001) in group of sleep before 23 o’clock of short sleep duration.

Since the collinearity of age, education, and current BMI was estimated (all tolerance >0.9 and VIF < 2), no variable was removed from the models. Therefore, a partial correlation analysis was performed to test the correlation between FS-14 scores and sleep duration in each group after being adjusted for age, years of education, and current BMI. There was a negative correlation of sleep duration with FS-14 physical score and total score in SBC of short sleep duration (*r* = −0.319, *p* = 0.001 and *r* = −0.315, *p* = 0.001, respectively; Figure 2).

## Discussion

This is the first study to investigate the association of sleep duration and bedtime with fatigue during the third trimester. The primary finding in the present study revealed that both having over 7 h of nighttime sleep and sleeping before 23 o’clock were indeed associated with low fatigue scores for pregnant women in the third trimester, as shown by lower physical and total scores of pregnant women in the third trimester in SBC of sleep duration >7 h than those in SBC of sleep duration ≤7 h and in SAC of sleep duration >7 h. In other words, sleeping less than 7 h or bedtime after 23 o’clock would increase the fatigue levels of pregnant women in the third trimester.

Sleep is necessary for the maintenance of good health and wellbeing, especially during pregnancy. Oregnant women are advised to obtain a minimum of 7 h of sleep during the third trimester (29). Many studies have focused on the topic of sleep of antepartum women in intensive care and medical-surgical units. Although pregnant women’s sleep characteristics in the hospital setting have been reported, very little is known about sleep in the third trimester of healthy pregnant women.

Short sleep duration due to late bedtime or early wake time or both is very common in a workday in modern society. Melatonin as an indoleamine plays a key role in the biological regulation of circadian rhythms, not only in mammals (30) but also in clinical evidence (31). It is well known that melatonin is a direct sleep-promoting effect hormone in diurnal species, and it is synthesized rapidly at night and released from the pineal gland directly into the bloodstream and the cerebrospinal fluid (32). Melatonin synchronizes and modulates circadian rhythms and related physiological functions *via* actions at G protein-coupled receptors, and melatonin receptor ligands modulate circadian rhythms (33). The temporal pattern of melatonin production is correlated with the sleep time of humans, and the onset of melatonin secretion is initiated approximately 2 h before an individual’s habitual bedtime and is correlated with the onset of evening sleepiness (34). Based on habitual sleep time at night, the sharp increase of melatonin usually occurs 2 h after the onset of its production (35). Furthermore, the melatonin rhythm in those following the late bedtime nights was delayed (36), while an abrupt imposition of bright light at night suppresses melatonin secretion (32). The administration of exogenous melatonin during the daytime also results in the induction of fatigue and sleepiness in humans (37). During pregnancy, maternal melatonin regulates maternal sleep and appears to pass unaltered into the fetus and entrain fetal circadian rhythms (38). Given the importance of sleep duration and bedtime in physiological processes, it is not surprising that disruption of circadian rhythms has numerous negative health consequences. Therefore, it is reasonable to arrange early bedtime (such as before 23 o’clock) and keep enough sleep duration (such as >7 h) to get as low a level of fatigue as possible, which is more significant and of particular concern in the third trimester of pregnancy.

The second finding demonstrated the negative correlation of sleep duration with FS-14 physical and FS-14 total score in the group of SBC with sleep duration ≤7 h, which suggested the importance of early bedtime. Optimal sleep is recognized for its positive contributions to physical performance and psychological wellbeing (39, 40). Sleep switches periodically between two markedly different states: non-rapid eye movement (NREM) sleep and rapid eye movement (REM) sleep, and NREM is subdivided into three stages based on a continuum from light sleep (Stage N1 and N2) to deep sleep (Stage N3). A complete sleep cycle (NREM and REM) of 90 min with cycling in this manner for 4–5 cycles per night is considered optimal. This first episode of NREM Stage N3 lasts approximately 10–30 min, which then gradually reduces to go into the cycle (41). It has been believed that sleep, especially slow-wave sleep (Stage N3), is vital for physical recovery due to its relationship with growth hormone release (42–44). Importantly, most N3 sleep occurs during the first half-night, whereas most REM sleep occurs during the second half-night (45). Therefore, early bedtime prolongs Stage N3 duration to decrease fatigue.

The average women’s habitual bedtime as reported was past midnight, but their rise time was confined due to work schedules (46). In a prospective study of nulliparous women with a singleton gestation, 40% of women reported short sleep duration (< 7 h sleep/night) in the third trimester (47). Further, since difficulties in breathing during sleep and in maintaining a conventional and everyday sleeping posture crop up during the period of late pregnancy and physical discomfort/physiological burden aggravate (48), an earlier bedtime should be encouraged for pregnant women, especially employed pregnant women, to increase the possibility of obtaining sufficient nocturnal sleep and to dissipate daily accumulated fatigue (46). In our present study, the negative correlation between sleep duration and FS-14 scores observed only in the group of SBC of sleep duration ≤7 h might be because the relationship became increasingly apparent when sleep was very deficient in this specific population (pregnant) and state (the third trimester). While for participants in the group of SAC, no matter if their sleep duration is ≤ 7 h or >7 h, because N3 sleep is vital for physical recovery and most N3 sleep occurs during the first half-night, their fatigue may be difficult to recover, making the relationship between sleep duration and fatigue scale difficult to observe.

Additionally, the association between sleep duration and bedtime with mental fatigue during the third trimester was not found in our present study. Mental fatigue has become a popular sub-healthy state nowadays (49). Mental fatigue represents a psychological state caused by prolonged periods of demanding cognitive activity (50). Too much brain activity and stimulation can make a person feel mentally tired, which is similar to physical fatigue (51). A previous study reported the decrease in maternal brain activity observed late in pregnancy (52). Moreover, REM sleep aids mental recovery (53) and most REM sleep occurs during the second half-night (45). This is consistent with our results, namely, no observed association between sleep duration and bedtime with mental fatigue during the third trimester. On the other hand, pregnant women who had the manifestation of some psychological states, such as self-reported depression, clinically appraised depression, anxiety disorders, state anxiety, and anxiodepressive comorbidities (54), had been excluded from our study. Therefore, to some extent, this study excluded the effect of sleep on mental fatigue in healthy pregnant women.

There are some limitations in this present study. First, data were collected from different weeks of the third trimester, but each data can represent the psychosomatic state of pregnant women during the whole third trimester. Second, the nulliparous and multipara pregnancy women were all included in the subjects recruited in this study, which might be considered as confounders when interpreting the results. Third, in the present study, since all participants did not complain or report their fatigue feeling before 28 weeks of pregnancy to gynecologists, we did not record baseline levels of fatigue or use continuous monitoring indicators. Finally, a larger sample size and measuring melatonin levels may further support our findings.

## Conclusion

Sleep less than 7 h or bedtime after 23 o’clock is associated with increasing fatigue levels of pregnant women in the third trimester. Therefore, it is necessary to develop good sleep habits (enough sleep duration and early bedtime) to keep fatigue at a low level for pregnant women in the third trimester.

## Data Availability Statement

The data that support the findings of this study are available from the corresponding authors. Requests to access these datasets should be directed to HL, huihli@pku.edu.cn.

## Ethics Statement

The studies involving human participants were reviewed and approved by the Institutional Review Board of the Second Affiliated Hospital of Xiamen Medical College. The patients/participants provided their written informed consent to participate in this study.

## Author Contributions

HL and FW designed the study. YK, DW, FW, and HL secured funding for the study. HL, DM, and DW led the drafting of the manuscript. HL led the statistical analyses. DM, HC, and LS collected the clinical data. YK, CS, and YL input the data. All authors approved the final manuscript for submission.

## Conflict of Interest

The authors declare that the research was conducted in the absence of any commercial or financial relationships that could be construed as a potential conflict of interest.

## Publisher’s Note

All claims expressed in this article are solely those of the authors and do not necessarily represent those of their affiliated organizations, or those of the publisher, the editors and the reviewers. Any product that may be evaluated in this article, or claim that may be made by its manufacturer, is not guaranteed or endorsed by the publisher.

## Abbreviations

SSD: short sleep duration
BMI: body mass index
FS-14: Fatigue Scale
ANCOVA: analysis of covariance
VIF: variance inflation factor
GLM: general linear models
SBC: sleep before 23 o’clock
SAC: sleep after 23 o’clock
SD: standard deviation.
